# Epidural Electrical Stimulation for Functional Recovery in Incomplete Spinal Cord Injury

**DOI:** 10.34133/cbsystems.0314

**Published:** 2025-07-22

**Authors:** Yihang Ren, Lifen Mo, Junlin Lu, Ping Zhu, Ming Yin, Wenqing Jia, Fengyan Liang, Xiaodi Han, Jizong Zhao

**Affiliations:** ^1^ Department of Neurosurgery, Beijing Tiantan Hospital, Capital Medical University, Beijing, China.; ^2^ China National Clinical Research Center for Neurological Diseases, Beijing, China.; ^3^ Sanya Research Institute of Hainan University, School of Biomedical Engineering, Hainan University, Sanya, China.; ^4^ State Key Laboratory of Digital Medical Engineering, Hainan University, Sanya, China.; ^5^ Department of Neurosurgery, West China Hospital of Sichuan University, Chengdu, Sichuan, China.

## Abstract

Epidural electrical stimulation (EES) has emerged as a promising treatment for spinal cord injury (SCI). However, the therapeutic potential of EES in functional recovery following incomplete SCI remains limited, with few studies of a large sample size. This study included 11 patients who received EES combined with physical therapy (PT) and 10 who received only PT. Follow-ups were conducted pre-surgery, post-surgery, and at 19 to 25 months postoperatively. After the surgery, patients in the EES + PT group showed significant improvements in sensory function (*P* < 0.001) and muscle spasticity (*P* < 0.001). Long-term follow-up indicated that the EES + PT group had significant improvements in sensory function (*P* < 0.001), muscle spasticity (*P* < 0.01), and urinary function (*P* < 0.05). Among them, all 11 patients had improvements in sensory function and muscle spasticity, and 6 of 11 reported an improvement in urinary function. Moreover, of the 5 patients with neuropathic pain, 4 exhibited reduced pain scores. Compared with the PT-only group, the EES + PT group had significantly better recovery in sensory function (*P* < 0.01), muscle spasticity (*P* < 0.0001), muscle strength (*P* < 0.01), and bowel function (*P* < 0.01). Further analysis suggested that patients with less severe SCIs in the EES + PT group tend to achieve better functional recovery. With a relatively large sample size compared to those in previous studies, this study confirms the promising therapeutic effects of EES in SCI. EES combined with PT provides a potential approach for functional recovery in patients with incomplete SCI.

## Introduction

Approximately 1 million individuals worldwide have paralysis due to spinal cord injuries (SCIs), with the number of new cases rising annually [1,2]. Following such injuries, patients typically experience reduced sensory and motor functions below the injury site [3–7], along with abnormalities in bowel [8,9], urinary [8,10,11], and sexual functions [8]. Complications following these injuries may include muscle atrophy, which peaks within the first 6 weeks following injury and continues thereafter [12,13]; muscle plasticity [14,15]; and heterotopic ossification, which is often associated with limited joint mobility and can lead to secondary disabilities in daily activities [16]. Additionally, there may be occasional occurrences of low blood pressure, arrhythmias, and abnormally low body temperature due to autonomic nervous system dysfunction [17,18]. Psychological and social challenges, such as stress, social isolation, and unemployment problems, may also occur [19,20].

Currently, the primary focus of SCI treatment is rehabilitation [11,21], aimed at enhancing patients’ quality of life and functional abilities. Traditional approaches, including medication, physical therapy (PT), and surgery, cannot fully reverse neural impairments caused by SCI. In recent years, epidural electrical stimulation (EES) has emerged as a promising rehabilitation method for SCI [6] (Table 1). Initially introduced by Melzack and Wall [22] in 1965 for chronic pain, EES has shown promise in modulating spinal cord circuits in animal models of SCI. Repeated training with EES can enhance motor control and enable individuals to regain voluntary control over paralyzed muscles [23–25]. Clinical studies supported EES’s role in functional recovery, including inducing spinal cord motor patterns for actions like standing and rhythmic movements [26,27]. EES works by implanting electrodes into the epidural space of the spinal cord to deliver electrical stimulation to neural circuits below the injury site. It enhances the excitability of neural networks, reactivating motor neurons and sensory pathways, which aids in the recovery of motor and sensory functions for paralyzed patients [28]. Repetitive stimulation and training can enhance SCI patients’ ability to control movements [29] and alter spinal cord excitability [30,31]. Rowald et al. [32] demonstrated that activity-specific EES rapidly enabled 3 SCI patients with complete sensorimotor paralysis to walk after surgery. Grahn et al. [33] reported a patient with chronic traumatic paraplegia who regained partial lower limb muscle function through EES of the lumbosacral spinal cord. However, understanding EES’s potential benefits in functional recovery following incomplete SCI remains limited, with few reported cases and unclear long-term effects. Most existing studies mainly focus on motor recovery [32,34]. Further, the existing studies cannot rule out the effects of PT, and the combined effects of EES and PT are largely unclear due to the lack of controlled studies. Thus, there is a need for cohort studies with relatively large-scale sample sizes to investigate the impact of EES on incomplete SCI to instruct clinical guidelines and inform practice.

**Table 1.** Recent studies on EES following SCI

Study	Sample size	ASIA grade	Physical therapy	Main outcomes	Study design
Harkema et al. [29]	1	B	√	Full-weight-bearing standing	Case report
Angeli et al. [28]	4	A (2); B (2)	√	Walking with a walker; weight-supported stepping; standing	Case series
Gill et al. [26]	1	A	√	Independent standing; walking with a walker; treadmill stepping	Case report
Rejc et al. [56]	4	A (2); B (2)	√	Full-weight-bearing standing	Case series
Kathe et al. [57]	9	A and B (3); C and D (6)	√	Weight-supported walking	Case series
Wagner et al. [38]	3	C (2); D (1)	√	Weight-supported walking	Case series
Darrow et al. [58]	2	A	×	Functional hypotension; urination	Case series
Herrity et al. [59]	10/10	A and B (10)	√	Urination	Controlled study
Zhang et al. [60]	2	B (1); C (1)	√	Walking with a walker	Case report
Romeni et al. [61]	2	C (2)	√	Walking recovery	Case report
This study	11/10	B (8); C (2); D (1)	√	Recovery in muscle strength, spasticity, and sensory	Cohort study

EES, epidural electrical stimulation; SCI, spinal cord injury; ASIA, American Spinal Injury Association

In this study, we included 11 patients who underwent implantation of stimulation electrodes in the thoracolumbar epidural space combined with PT and 10 patients who received PT alone. We aimed to comprehensively investigate the therapeutic effects of EES on incomplete SCI in terms of sensation, muscle strength, spasticity, and autonomic function. We aimed to derive accurate treatment estimates and to explore potential factors influencing follow-up outcomes. Additionally, we compared the combined effects of EES and PT with those of PT alone. To the best of our knowledge, this is the first cohort study of EES in China involving a relatively large number of SCI individuals.

## Methods

### Ethics approval

The study was approved by the Ethics Committee of Beijing Tiantan Hospital, Capital Medical University, on 2021 February 9 (Approval No. KY 2021-012-02). All participants were fully informed and provided their written consent to participate in the research prior to their involvement. The authors declare that all procedures comply with relevant laws and institutional guidelines and have been approved by the appropriate institutional committee.

### Data sources and participants

**Fig. 1.**
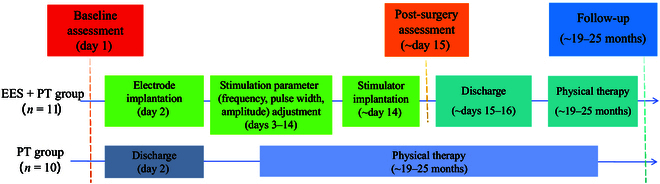
Study design and timeline of the EES + physical therapy (PT) group and the PT-only group.

We conducted a nonrandomized, nonblinded, controlled study at Tiantan Hospital from October 2020 to April 2024. The study included all patients over 18 who met the specified inclusion criteria. Patients with an American Spinal Injury Association (ASIA) grade ≥B were eligible for inclusion; this grade indicates a loss of motor function below the level of injury but with some preservation of sensory function. The level of injury had to be above the L1 and L2 vertebrae. To ensure that the study observed the treatment effects during the chronic phase, patients had to have been at least 6 months postinjury, excluding the rehabilitation effects during the acute and subacute phases. Priority was given to patients with acute traumatic injuries, particularly those resulting from accidents and falls from heights (Fig. 1).

### Procedure

Data on demographic characteristics, clinical features, and radiological examinations were extracted from the electronic medical recording system. Demographic characteristics included age, sex, etiology, and time since injury. Clinical features included assessment of limb muscle strength, muscle spasticity, sensation, bladder and bowel functions, and pain perception. Radiological examinations included magnetic resonance imaging (MRI), electromyography/motor somatosensory evoked potentials (EMG/SEP), and computed tomography (CT) scans.

All patients (*n* = 21) underwent preoperative 1.5-T MRI scans to obtain sagittal and axial images of the spinal cord at the injury site. These images were used to assess the location of the lumbar enlargement and guide electrode implantation. Additionally, CT scans of the lumbar enlargement were conducted to rule out the risk of spinal cord compression. EMG/SEP and urodynamic electrophysiological studies were performed on all patients to evaluate autonomic nerve functions, focusing primarily on the lower limbs and bladder and bowel functions, to exclude patients with complete SCI. The baseline neurological function was assessed by ASIA [35], the Modified Ashworth Spasticity Scale [36], the Bladder and Bowel Function Scale, and the Visual Analog Scale for Pain during the preoperative period [37]. After preoperative evaluation, 21 patients met the criteria for implantation. Among them, 11 patients agreed to undergo EES treatment, while 10 opted for conservative treatment. All 21 patients received PT under the guidance and recommendations of their physicians. We contacted the patients and their families on a regular basis to provide remote guidance and support. This treatment included lower limb functional training, rehabilitation cycling, quadricep training, ankle joint continuous passive motion training, and reduced-weight treadmill training. The daily training duration was 4 to 5 h.

### EES surgery

**Fig. 2.**
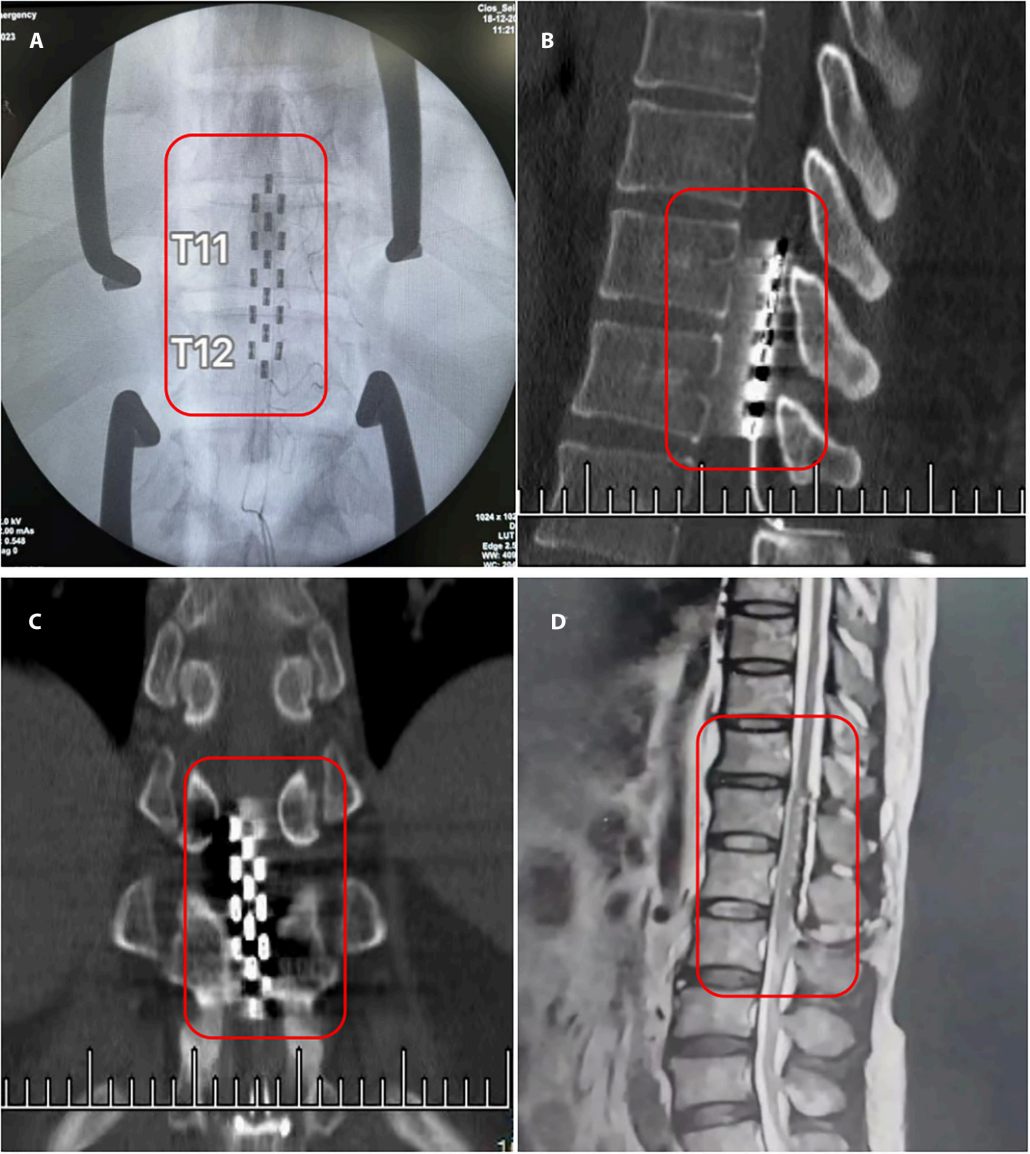
(A) Intraoperative x-ray positioning, taking an example of implantation at T11 to T12. Postoperative computed tomography (CT) in the sagittal (B) and coronal (C) views. (D) Postoperative magnetic resonance imaging (MRI) in the sagittal view.

**Fig. 3.**
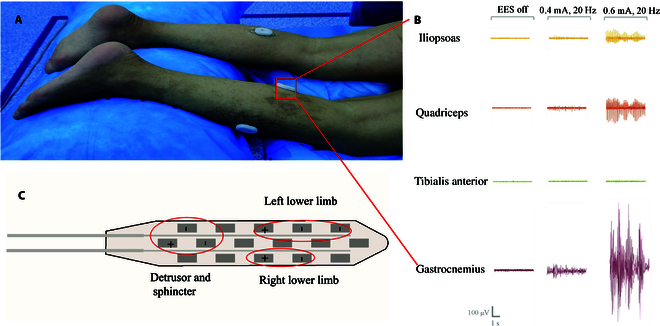
(A) The surface electromyography (EMG) system applied to the target muscles of both lower limbs of the patient (iliopsoas, quadriceps, tibialis anterior, gastrocnemius, and soleus). (B) Intraoperative EMG recordings from the iliopsoas, quadriceps, tibialis anterior, and gastrocnemius muscles under 3 conditions: EES is off, stimulation at 0.4 mA and 20 Hz, and stimulation at 0.6 mA and 20 Hz. (C) Example of electrode configuration in one patient, covering the spinal cord areas of the bilateral proximal and distal muscles, detrusor, and sphincter (caudal positions). The evaluation of the detrusor and sphincter was conducted postoperatively.

The patients in the intervention group initially underwent implantation of a spinal cord electrode (model 39565, Medtronic) and a temporary EES stimulator (model 37714, Medtronic). Under general anesthesia, patients were positioned prone. The implantation process was tailored to the anatomical location of the lumbar enlargement. For example, if the lumbar enlargement was located at T11 to T12, the following steps were taken:

1. The T12 to L1 spinous processes were identified using fluoroscopy, and an incision of approximately 3 to 5 cm was made along the midline, exposing the interspinous space.
2. The soft tissue in the space was cleared, and part of the vertebral lamina was excised to create an approximately 2-cm-wide opening, allowing direct visualization of the dura mater.
3. A rigid electrode sample, provided together with the electrode, was used to expand the epidural space. It is crucial to avoid blood vessels, as this can prevent increased electrode impedance or spinal cord compression.
4. After confirming the position, the sample was removed, followed by the implantation of the stimulation electrode. A C-arm x-ray scan was conducted to verify the electrode’s position (Fig. 2A).
5. Intraoperative electrophysiological monitoring guided the physician in selecting appropriate stimulation parameters (frequencies, pulse widths, amplitude, and anode and cathode configuration) and finalizing the vertical and lateral positions. This process typically lasts 0.5 to 1 h to ensure that the stimulation range encompasses the target muscle groups of both the left and right lower limbs (Fig. 3). Once the coverage range was confirmed, the electrode base was secured to the interspinous space with a titanium plate to prevent displacement.
6. A longitudinal incision, 2 to 4 cm in length, was made lateral to the initial incision, and the lead was passed through the subcutaneous fat layer at this site.
7. After connecting the extension lead, the impedance of the electrode was tested. Once it met the impedance requirements, the extension lead was tunneled through the subcutaneous fat layer to emerge from the dorsal skin.
8. The electrode was then fixed at this site to prevent inward sliding and potential infection.
9. A postoperative CT and MRI reexamination was conducted (Fig. 2B to D) to finalize the verification of the electrode’s position.

After achieving a positive outcome or confirming no adverse effects in the patient’s clinical assessment after 7 to 10 d, we proceeded with the implantation of the implantable stimulator (model 37714, Medtronic). The stimulator was implanted into the subcutaneous fat and muscular fascial space in the upper right quadrant of the abdomen. The extended lead from the back incision was then removed, and a subcutaneous tunnel was fashioned to connect the original lead to the stimulator. Then, the electrode impedance and the stimulator functions were tested. Upon successful confirmation, the stimulator was anchored to the fascia using nonabsorbable sutures.

### Configuration of EES stimulation protocols

The configuration of the temporary stimulation protocol begins 1 d after surgery. The parameters include anode and cathode configuration, amplitude, pulse width, and frequency. The electrode configuration is guided by preoperative assessment to ensure coverage of the 5 major muscle groups (iliopsoas, quadriceps femoris, tibialis anterior, gastrocnemius, and soleus) in the lower limbs. This study primarily focuses on movement and muscle spasticity, so low-frequency stimulation (typically around 20 Hz) is preferred. The pulse width is adjusted based on the patient’s spinal cord thickness, generally at 220 μs [26,33]. The EES was kept on continuously all day. The voltage was tailored to the patient’s subjective perception, with settings just below the threshold for subtle muscle twitching, typically around 3 V (or 3 mA in the current mode). The tolerance voltage varied among different patients. In some cases, patients could tolerate voltage of up to around 9 V. Additionally, we established a set of stimulation parameters targeting the detrusor and sphincter muscles based on the patient’s reported sensation of perineal tingling. After the surgery, the stimulation frequency and pulse width for each patient were determined and could not be modified by the patients themselves for safety reasons. However, patients were allowed to adjust the stimulation voltage based on their individual tolerance and feedback. During the follow-up, no electrode-related complications (such as infection or displacement) were observed in any of the 11 patients.

After determining the postoperative temporary stimulation protocol, clinical assessments were conducted based on preoperative assessment scales and patient self-reported experiences. If the patients exhibit sensory recovery below the level of the lesion, improved muscle strength, or reduced muscle spasticity, permanent stimulator implantation is considered. The evaluation process usually lasts 7 to 10 d.

### Outcome assessment

The neurological function was assessed at 3 time points, baseline (admission), post-EES surgery (within 14 d postoperatively), and follow-up (19 to 25 months), to evaluate the efficacy of EES treatment. The data collected included indicators such as sensory function, muscle strength, spasticity, urinary control, bowel function, and chronic pain. ASIA sensory scores (C2 to S5) were categorized as normal, decreased, or absent, with a total possible score of 224. The ASIA motor score focused on the 5 muscle groups of the lower limbs (L2 to S1). Muscle spasticity was assessed using the Modified Ashworth Spasticity Scale. Urinary control was assessed based on the awareness of bladder emptying, the ability to prevent urinary leakage, and bladder-emptying methods. Bowel function was evaluated based on the sensation of needing to defecate, the ability to prevent stool leakage (bowel control), and voluntary contraction of the sphincter. Pain was measured using the Visual Analog Scale.

### Statistical analysis

Statistical analyses were performed using SPSS V.29 (IBM, USA) and Prism V.10 (GraphPad Software, USA) to evaluate the results, assess the efficacy of EES treatment, and identify related factors contributing to the EES benefits. The normality of data distribution was assessed using the Kolmogorov–Smirnov test. For continuous variables, data with a normal distribution are presented as mean ± standard deviation (SD) and were compared using *t* tests. Nonnormally distributed data are presented as median and compared using the Wilcoxon rank-sum test. Categorical variables were compared using the χ^2^ test. Two-tailed *P* values <0.05 were considered statistically significant. Outcomes were compared between the EES + PT group and the PT-only group to analyze the efficacy of EES treatment. Additionally, a univariate analysis of factors was conducted to identify potential factors associated with neurological improvement.

## Results

### Baseline characteristics

We identified 21 eligible participants with incomplete SCI for electrode implantation in the lumbar epidural space (Table 2). The mean age was 44.4 years (SD, 18.6 years), with a male-to-female ratio of 2:1. Among them, 11 patients underwent permanent stimulation electrode implantation with a neuromodulation program and PT, while 10 chose a nonsurgical treatment option (PT only). Follow-up outcomes were collected for all 21 patients.

**Table 2.** Treatment groups and clinical characteristics of patients

Variables	Treatment groups		*P* value
	EES + PT (*n* = 11)	PT (*n* = 10)	
Age, years, mean (SD)	48.7 (18.1)	39.7 (18.8)	0.277
Gender (%)			0.183
Male	9 (80.0)	5 (50.0)	
Female	2 (20.0)	5 (50.0)	
Time since injury, months, mean (SD)	25.8 (6.5)	30.6 (14.7)	0.338
Time since enrollment, months, mean (SD)	21.6 (1.9)	22.7 (2.0)	0.228
ASIA grade (%)			0.455
B	8 (72.7)	6 (60.0)	
C	2 (18.2)	1 (10.0)	
D	1 (9.1)	3 (30.0)	
Level of injury (%)			0.260
Cervical	7 (63.6)	4 (40.0)	
Thoracic	4 (36.4)	6 (60.0)	
ASIA sensation score, mean (SD)	127 (43.6)	122 (34.0)	0.329
ASIA motor score, median (IQR)	0 (0–12.0)	0 (0–33.0)	0.666
Modified Ashworth Spasticity Scale, median (IQR)	26.0 (22.0–28.0)	19.5 (5.5–22.3)	0.070
Urinary function score, median (IQR)	1 (0–1)	1 (0–2.3)	0.751

Time since injury: the time from injury to surgery. Time since enrollment: the time from enrollment to follow-up. Level of injury: cervical refers to injuries in the cervical spine (C4 to C7), and thoracic refers to injuries in the thoracic spine (T4 to T11). IQR, interquartile range

The preoperative time (from injury to surgery) for the EES + PT group and the PT-only group was 25.8 ± 6.5 and 30.6 ± 14.7 months, respectively. The follow-up duration (from enrollment to follow-up) for the EES + PT group and the PT-only group was 21.6 ± 1.9 and 22.7 ± 2.0 months, respectively. The ASIA grades at enrollment (B, C, and D) showed no significant difference between the EES + PT and PT-only groups. Similarly, the 2 groups had no significant difference in the level of injuries. Overall, the clinical characteristics of the patients in both groups did not show significant differences (*P* > 0.05) (Table 2).

The ASIA sensation scores for the EES + PT and PT-only groups were 127 ± 43.6 and 122 ± 34.0, respectively (*P* > 0.05). The ASIA motor scores had a median (interquartile range [IQR]) of 0 (0 to 12.0) in the EES + PT group and 0 (0 to 33.0) in the PT-only group (*P* > 0.05). Muscle spasticity, assessed by the Modified Ashworth Spasticity Scale, had a median (IQR) of 26.0 (22.0 to 28.0) in the EES + PT group and 19.5 (5.5 to 22.3) in the PT-only group (*P* > 0.05). Urinary function scores were reported as a median (IQR) of 1 (0 to 1) in the EES + PT group and 1 (0 to 2.3) in the PT-only group (*P* > 0.05) (Table 2).

**Fig. 4.**
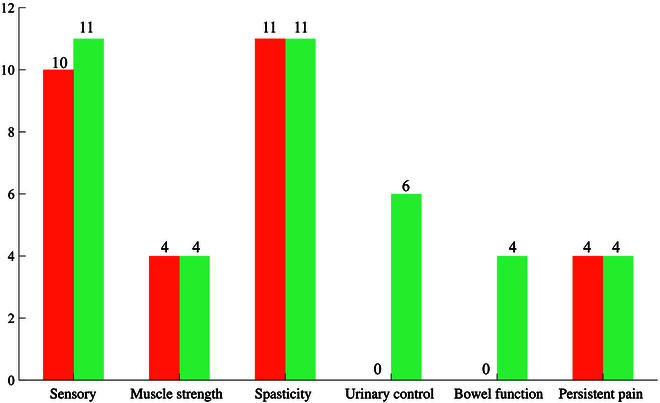
Short-term (in orange) and long-term (in green) functional outcomes of 11 patients receiving EES and PT. The number represents the number of patients with improved outcomes.

**Fig. 5.**
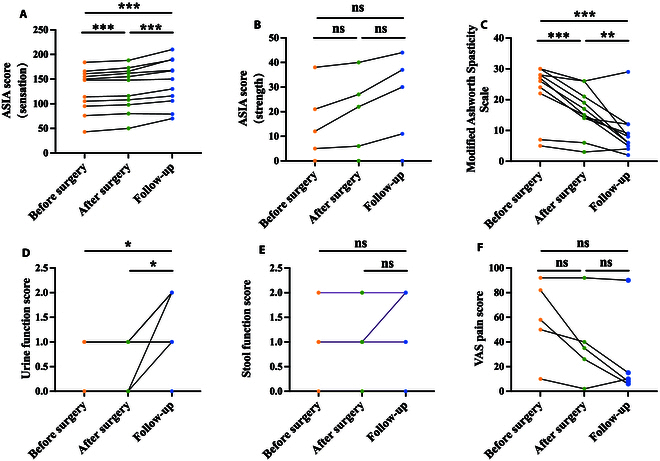
Functional outcomes of the EES + PT group over 3 periods. (A) Sensation (*n* = 11); (B) strength (*n* = 11); (C) muscle spasticity (*n* = 11); (D) urine function (*n* = 11); (E) stool function (*n* = 11); (F) pain (*n* = 5) (*P < 0.05; **P < 0.01; ***P < 0.001; ns, not significant). VAS, Visual Analog Scale.

The short-term efficacy of EES treatment was quantified by comparing the pre- and postoperative functional outcomes of the EES + PT group (Figs. 4 and 5). Note that the postoperative outcome assessment was performed on the 14th postoperative day. During this period, patients were confined to bed and did not receive PT.

EES demonstrated short-term therapeutic effects on SCI patients in various aspects. Before surgery, all patients exhibited spastic paralysis and abnormalities in sensation (light touch and pinprick), accompanied by sensory impairment below the level of injury. After surgery, muscle spasticity improved in all 11 patients, and sensation recovered in 10 patients. These improvements were statistically significant (*P*< 0.001). Moreover, 4 of 11 (36.4%) patients improved lower limb strength Among the 5 patients with intractable neuropathic pain, 4 (80%) exhibited reduced pain scores. However, no significant changes were observed in strength scores urinary and stool functions and pain scores after the surgery.

### Long-term efficacy of EES and PT treatment

The long-term efficacy of EES and PT treatment was quantified by comparing the preoperative and long-term follow-up outcomes of the EES + PT group (Figs. 4 and 5). Significant improvements were observed in sensation (*P* < 0.001), spasticity (*P* < 0.001), and urine function scores (*P* < 0.05) during long-term follow-up. All 11 patients exhibited recovery in sensation and spasticity, while 6 of 11 (54.5%) patients exhibited recovery in urine function. What is more, 4 of 11 (36.4%) patients showed improvement in lower limb strength, 4 of 5 (80%) patients with neuropathic pain exhibited reduced pain, and 4 of 11 (36.4%) patients showed improvement in stool function.

### Neurological function recovery of EES + PT and PT treatment

**Fig. 6.**
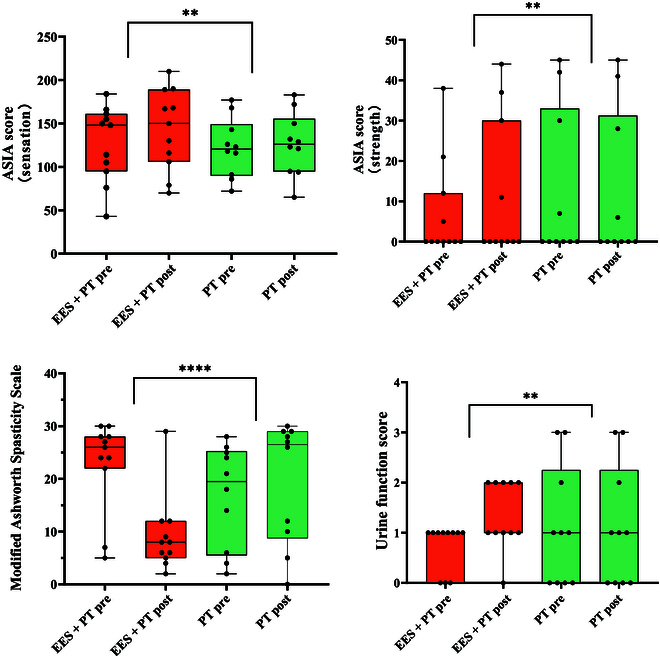
Sensation, strength, muscle spasticity, and urine functional outcomes of the EES + PT (*n* = 11) and PT-only (*n* = 10) groups at the baseline (pre) and after the treatment (post, 19 to 25 months).

We compared the long-term functional recovery between the EES + PT group and the PT-only group (Table 3). The results demonstrated that the EES + PT group showed significantly greater improvements in sensory function (*P* < 0.01), muscle spasticity (*P* < 0.0001), muscle strength (*P* < 0.01), and bowel function (*P* < 0.01) compared to the PT-only group (Fig. 6).

**Table 3.** Comparison of the long-term functional recovery between the EES + PT and PT-only groups

Functional recovery	EES + PT (*n* = 11)			PT (*n* = 10)		
	Positive	Unchanged	Positive rate(%)	Positive	Unchanged	Positive rate(%)
Sensation	11	0	100	9	1	90.0
Strength	4	7	36.4	0	10	0
Muscle spasticity	11	0	100	2	8	20.0
Urine function	6	5	54.5	0	10	0
Stool function	4	7	36.4	1	9	10.0

Positive: there is an improvement in the scale scores. Unchanged: there is no improvement in the scale scores

First, in terms of sensory function, the EES + PT group exhibited a significant improvement in ASIA scores compared to the PT-only group (*P* < 0.01). The sensory recovery rate in the EES + PT group (100%) was also higher than in the PT-only group (90.0%). Second, muscle strength, as measured by ASIA motor scores, was significantly enhanced in the EES + PT group compared to that in the PT-only group (*P* < 0.01). No patients in PT-only group had improvement in muscle strength. Third, muscle spasticity, evaluated using the Modified Ashworth Spasticity Scale, demonstrated a significant reduction in the EES + PT group compared to that in the PT-only group (*P* < 0.0001). The recovery rate of muscle spasticity in the EES + PT group (100%) is also higher than that in the PT-only group (20.0%). Lastly, urinary control also showed significant improvement in the EES + PT group (*P* < 0.01), while no patients in the PT-only group recovered in urinary control. However, no statistically significant differences were observed in bowel function recovery between the 2 groups, although the recovery rate of the EES + PT group (57.1%) is much higher than that of the PT-only group (11.1%).

Therefore, EES combined with PT significantly outperformed PT alone in restoring sensory function, muscle strength, spasticity, and urinary function. These results underscore the advantages of combining EES with PT to enhance neurological recovery in patients with SCI.

### Factors associated with therapeutic effects

Univariate analysis was used to investigate the potential factors associated with treatment efficacy (Table S1). Sensory and muscle spasticity were not analyzed, since all patients in the EES + PT group had long-term recovery in sensory and muscle spasticity. The following potential factors were considered: age, gender, time since injury, injury etiology, injury site, injury grade, and postoperative recovery duration. Results indicated that patients with mild injuries (ASIA C to D) demonstrated significant improvements in muscle strength compared to those with severe injuries (ASIA B) (*P* < 0.05). This suggests that SCI patients with ASIA C to D tend to achieve greater recovery in muscle strength than those with ASIA B. However, no other factors significantly influence the prognosis.

### Representative cases in the EES + PT group

**Fig. 7.**
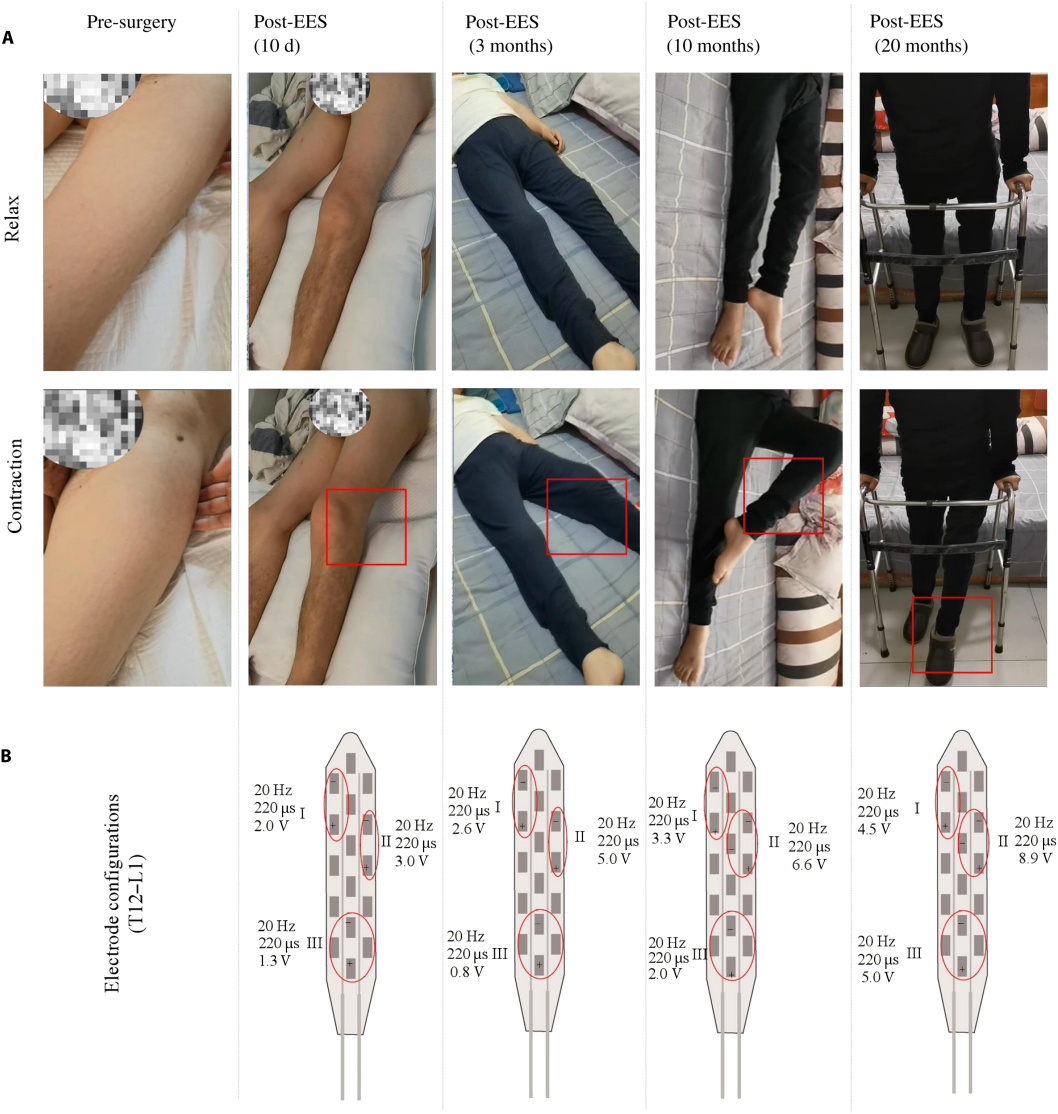
(A) One representative patient (P1) performed muscle contractions before and after EES surgery at different times. The squares highlight the largest movement of the knee or ankle joints. After 20 months, the patient can walk with a walker while EES is on. (B) Electrode configurations (including positions, frequencies, amplitude, and pulse width) were adjusted according to the patient’s feeling and performance at different times. Groups I, II, and III (red circles) are the configurations for the left lower limb, right lower limb, and detrusor and sphincter, respectively.

We selected 2 patients from the EES + PT group as examples (Table 4 and Fig. 7) to demonstrate the recovery process. All 2 patients could walk with a walker at the time of follow-up. For patient P1, who sustained a T11 to T12 SCI classified as ASIA C, ASIA motor function scores improved from 5 to 13 points after 19 to 25 months of treatment. This improvement was particularly evident in lower limb motor scores, with gains in L2 and L3 muscle strength. Additionally, P1’s ASIA sensory score increased from 166 to 189.

**Table 4.** Two representative patients (P1 and P2) following EES combined with PT. Patient characteristics and functional outcomes were assessed before surgery and during follow-up (19 to 25 months post-surgery).

Patient	P1		P2	
Gender	M		M	
Age	36		74	
Months after SCI	28		17	
Level of injury	T11–T12		C4	
Pre (before surgery); post (19–25 months post-surgery)	Pre	Post	Pre	Post
ASIA grade	C	C	C	D
Lower extremity motor scores				
L2 (left|right)	3|0	3|2	1|2	3|3
L3 (left|right)	1|1	4|3	1|2	2|3
L4 (left|right)	0|0	1|0	1|2	2|3
L5 (left|right)	0|0	0|0	1|2	2|3
S1 (left|right)	0|0	0|0	1|2	2|3
Total (total: 50)	5	13	18	31
ASIA sensory score (total: 224)	166	189	114	130

Similarly, patient P2, with a cervical-level injury (C4) and classified as ASIA C, achieved considerable functional recovery. His lower limb motor scores improved from 18 to 31, with notable gains in L2 strength. The sensory score for P2 also increased from 114 to 130 points during the follow-up (Table 4). Fig. 7A illustrates the improved limb movement of P1 over time, with marked recovery observed as early as 40 d and sustained improvements up to 20 months post-surgery (Movie S1). The electrode positions (Fig. 7B) targeted specific spinal regions responsible for motor and sphincter control, suggesting that precise electrode positioning contributes to the observed functional recovery. These findings underscore the potential of EES combined with PT in promoting long-term recovery in motor and sensory functions in patients with different SCI levels.

## Discussion

We conducted a cohort study with a relatively large sample size, comprehensively analyzing the efficacy of EES and comparing it with that of traditional PT. We assessed the impact of this treatment across various aspects, including sensory and motor functions, muscle spasticity, urinary and bowel functions, and intractable pain. This study highlights the promise of EES combined with PT to promote sensory recovery in patients with incomplete SCI.

This study established strict inclusion criteria to ensure accurate assessment of the effects of EES, with an age limit of 18 years, and only patients with an injury duration of over 6 months and ASIA scores ≥B were included. These conditions were set to minimize potential complications and ensure a more controlled environment for evaluating treatment outcomes. However, future research could consider broadening these criteria, such as including younger patients or those in the acute phase, to examine the efficacy of EES in a broader range of SCI conditions. In this study, we set an age limit of 18 years for inclusion, as patients under 18 may still experience rapid physical development, mainly due to the differential growth between the spinal cord and spine. Clinical assessment of injury severity relied on ASIA grading based on clinical manifestations of SCI. Patients with sensory function (≥ASIA B) below the level of injury can provide valuable feedback about their sensations (comfort/discomfort), which aids in the adjustment of EES parameters. Thus, we selected patients with ASIA scores ≥B. Regarding the level of injury, we opted for implantation at the lumbar enlargement based on clinical observations and previous research highlighting the importance of intact neural circuits for lower limb stimulation [28,38]. In our study, the patient’s injury range spanned C4 to T12. The experiment primarily included patients with acute traumatic injuries. During clinical observations, we noted that within the initial 6 months postinjury, patients tended to experience partial functional recovery. However, the recovery process gradually entered a plateau phase with a markedly slower improvement rate. To mitigate the potential risk of spinal cord compression due to complications such as spinal cord swelling and adhesions, especially in the acute and subacute phases following electrode implantation, we only included patients with an injury duration of over 6 months.

EES combined with PT demonstrated superior efficacy compared to traditional PT treatment. In previous EES studies, SCI patients often receive PT at the same time, and the effects of PT cannot be ruled out. With a relatively large sample size, the EES + PT and PT-only groups in this study had no significant differences in clinical characteristics or injury duration (Table 2). However, long-term follow-up indicated that the EES + PT group exhibited significantly improved sensation (*P* < 0.01), muscle strength (*P* < 0.01), muscle spasticity (*P* < 0.0001), and urinary function (*P* < 0.01) compared to the PT-only group, as determined by one-way analysis of variance. Thus, EES combined with PT is strongly recommended for SCI patients to achieve better functional recovery. Previous studies also suggest that PT is essential after EES surgery to enhance neurological function recovery [26,39,40].

EES combined with PT significantly improved short- and long-term sensory function in patients with SCI. Sensory function showed significant improvement in all 11 patients who received EES + PT treatment during both short- and long-term follow-up (*P* < 0.001, *P* < 0.001). Notably, sensory recovery in the EES + PT group was significantly better than that in the PT-only group. These results suggest that EES enhances neural plasticity and sensory pathways, offering a valuable therapeutic approach for sensory recovery in SCI patients. Restoration of pain–temperature perception, gross touch, and pinprick sensation helps prevent injuries such as burns, frostbite, and pressure ulcers, which may result from abnormal sensations [41]. This also facilitates the early initiation of rehabilitation training in patients without infection or skin damage. EES after SCI may promote sensory recovery through several mechanisms. It enhances neuroplasticity by reactivating neural networks below the injury site and facilitating synaptic remodeling. Additionally, electrical stimulation activates residual neural pathways, thereby improving the excitability of spinal cord circuits, which aids sensory transmission. It may also promote nerve fiber regeneration and enhance coordination between sensory and motor pathways, fostering a more integrated recovery of sensory and motor functions. These processes are particularly significant in the early recovery stages post-SCI [28,42–44].

In our study, 4 of 11 patients in the EES + PT group experienced an increase in muscle strength, compared to none in the PT-only group. This improvement is statistically significant compared to the PT group (*P* < 0.01). Additionally, patients’ feedback suggests that EES + PT alleviates their psychological burden and enhances lower limb motor function. Additionally, the result of the univariate analysis indicates that the ASIA grade was significantly correlated with the recovery of muscle strength (*P* < 0.05). Patients with less severe injury (ASIA C to D) tend to achieve better recovery in muscle strength.

**Fig. 8.**
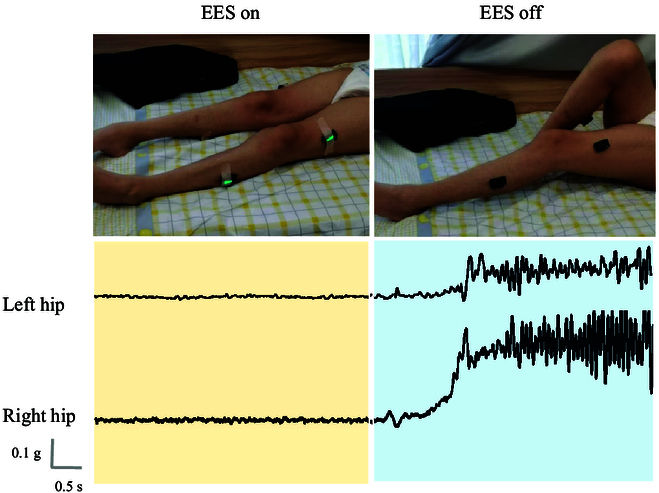
The accelerations in the upward direction measured by sensors at the thighs of one representative patient with muscle spasticity: EES can reduce the muscle spasticity. When EES is turned off, muscle contraction occurs unnaturally, as evidenced by a rapid increase in the acceleration in the upward direction at the thighs.

EES combined with PT significantly improved both short- and long-term muscle spasticity in patients with SCI. This study primarily focused on 4 muscle groups in the lower limbs: the iliopsoas, quadriceps femoris, tibialis anterior, and gastrocnemius, which correspond to the movement of the hip, knee, and ankle joints. We found that the therapeutic effects on muscle spasticity disorders were more significant in the EES + PT treatment group compared to those in the PT-only group (*P* < 0.0001). Previous studies [45,46] also suggest that EES treatment holds substantial clinical significance for patients with muscle spasticity, particularly those with SCI. In the EES + PT group, reduced muscle spasticity was observed after EES surgery and persisted with continuous stimulation without symptom recurrence (Fig. 8 and Movie S2). The reduction in muscle spasticity in patients treated with EES + PT was evident in the short term and continued throughout the treatment period. Overall, EES combined with PT treatment significantly alleviates muscle spasticity induced by SCI, aiding patients’ lower limb movement. However, we also observed that when electrical stimulation is turned off, patients experience a rebound in muscle spasticity (Fig. 8) and cannot disengage. This requires further investigation into its mechanisms and a more extensive study and analysis with a larger sample size.

EES combined with PT significantly improved long-term urinary control and bladder function in patients with neurogenic bladder (NB). In recent years, EES has emerged as a promising treatment for NB [47]. Studies in rats targeting the lumbar and sacral regions of the spinal cord have elucidated the neural regulation of the detrusor muscle and external urethral sphincter [48]. Research confirms that stimulation at the upper lumbar vertebrae (L1) and lower lumbar-sacral vertebrae (L5 to S6) primarily activates the detrusor muscle, while sacral stimulation predominantly activates the external urethral sphincter [48–50]. We found that stimulation from the lower thoracic to upper lumbar segments also contributes to the recovery of NB. Significant differences in bladder function recovery were observed between the EES + PT group and the PT-only group (*P* < 0.01), indicating the therapeutic effect of EES treatment on restoring urinary function. In the EES + PT group, we observed no significant changes in urinary function during hospitalization. However, improvements in urinary function were identified during the long-term follow-up, indicating that urinary function recovery requires an extended period. Thus, this therapy contributes to renal function protection, enhanced quality of life, and urinary catheter removal, ultimately restoring normal urinary function. This approach complements sacral nerve stimulation therapy for patients with NB accompanied by impaired lower limb function.

EES treatment alleviated neuropathic pain in patients with SCI, with 80% of those experiencing pain reporting relief. Following SCI, some patients may experience intractable neuropathic pain, which often remains uncontrolled with conventional drug treatments. EES has emerged as a widely used treatment for various chronic pain conditions that are resistant to medication [51–53]. In our study, 5 of 11 patients undergoing EES treatment reported the emergence of pain. The pain locations included 2 cases of intercostal back pain, 1 case of perineal discomfort, and 2 cases of lower limb pain (predominantly in the toes). Among these patients, 4 (80%) experienced pain relief. Further research is needed due to the limited sample size of patients with pain in this study.

### Limitations and prospects

As a single-center prospective study, our study may be subject to patient selection bias. An optical motion capture system with force plates can provide useful kinematic data (e.g., joint angles, velocities, and step length) and dynamic data (e.g., torque and ground reaction force) to better quantify patients’ functional recovery. To optimize the study protocol, future research should expand the sample size to enhance statistical power and generalizability. Stratified analysis based on injury severity (ASIA grades B, C, and D) would provide more specific insights into the effects of EES combined with PT. The variety in rehabilitation contents among patients may be a weakness, although we maintained regular remote contact and follow-ups with the patients. Single-center studies that control the rehabilitation contents and intensities will be conducted in the future. More extended follow-up periods are necessary to assess the long-term sustainability of recovery. Incorporating objective sensory measurements, such as electrophysiological measurement, and exploring neuroplasticity mechanisms will enhance understanding. Controlling for confounding factors such as age and comorbidities and comparing EES + PT with other interventions will validate its efficacy. Lastly, tailoring treatment protocols based on individual patient needs could maximize therapeutic outcomes.

In recent years, the brain–spine interface [34,54] has shown promise as a supplement for patients with SCI. The brain–spine interface integrates the brain–computer interface (BCI) and neuromodulation technologies and establishes a digital bridge between the brain and the spinal cord, involving the user’s intentions to control the EES in real time to meet daily needs [34,55]. It is believed that BCI and EES will benefit patients with SCI in the future.

## Conclusion

Previous EES studies have shown promise in SCI patients, but they cannot rule out the effects of PT. This study comprehensively evaluated the combined effects of EES and PT on sensory, motor, and autonomic functions in SCI patients. The results indicate that EES combined with PT significantly enhances long-term sensory function, muscle strength, spasticity, and urinary function in SCI patients compared to PT alone. The combined treatment also improved bladder control and pain relief in some patients. Further research with larger cohorts, longer follow-ups, and objective assessments is needed to optimize treatment protocols and confirm the long-term efficacy of this approach. Emerging technologies like BCI may further enhance SCI rehabilitation, offering promising prospects for personalized treatment.

## Data Availability

The data that support the findings of this study are available on reasonable request from the corresponding authors.
