# Supplementary material for: Epidural Electrical Stimulation for Functional Recovery in Incomplete Spinal Cord Injury
Source: Cyborg Bionic Syst. 2025 Jul 22;6:0314. doi: 10.34133/cbsystems.0314 (PMC12280331; doi:10.34133/cbsystems.0314)
Supplement: Supplementary 1 — Table S1 Movies S1 and S2 [file cbsystems.0314.f1.zip › Table S1.docx]

**Table 4. Univariate analysis of risk factors associated with improvement in neurological functions.**

|  | All patients (n=11) | Therapeutic effect | | | | | | | | |
| --- | --- | --- | --- | --- | --- | --- | --- | --- | --- | --- |
|  |  | Strength | | | Urine function | | | Stool function | | |
|  |  | Positive | Unchanged | p value | Positive | Unchanged | p value | Positive | Unchanged | p value |
| Sex |  | | | | | | | | | |
| male | 9 | 2 | 7 | 0.109 | 3 | 6 | 0.182 | 2 | 7 | 0.109 |
| female | 2 | 2 | 0 |  | 2 | 0 |  | 2 | 0 |  |
| Age (years) |  | | | | | | | | | |
| 18-48 | 5 | 2 | 3 | 1 | 3 | 2 | 0.567 | 3 | 2 | 0.242 |
| >48 | 6 | 2 | 4 |  | 2 | 4 |  | 1 | 5 |  |
| Time since injury (months) |  | | | | | | | | | |
| 16-25 | 5 | 2 | 3 | 1 | **2** | 3 | 1 | 3 | 2 | 0.242 |
| 26-35 | 6 | 2 | 4 |  | 3 | 3 |  | 1 | 5 |  |
| Site of injury |  | | | | | | | | | |
| C | 4 | 1 | 3 | 1 | 1 | 3 | 0.545 | 1 | 3 | 1 |
| T | 7 | 3 | 4 |  | 4 | 3 |  | 3 | 4 |  |
| ASIA grade |  | | | | | | | | | |
| B | 8 | 1 | 7 | 0.024 | 2 | 6 | 0.061 | 2 | 6 | 0.491 |
| C-D | 3 | 3 | 0 |  | 3 | 0 |  | 2 | 1 |  |
| Post-operative (months) |  | | | | | | | | | |
| 19-21 | 5 | 2 | 3 | 1 | 2 | 3 | 1 | 1 | 4 | 0.348 |
| 22-25 | 6 | 2 | 4 |  | 2 | 4 |  | 3 | 3 |  |

Due to the limited sample size, Fisher's exact test was employed as the conditions for Pearson chi-square test were not met.
